# Disability-adjusted life years for acute bronchiolitis in infants in Colombia

**DOI:** 10.11604/pamj.2021.39.236.25761

**Published:** 2021-08-12

**Authors:** Jefferson Antonio Buendía, Diana Guerrero Patiño

**Affiliations:** 1 Grupo de Investigación en Farmacología y Toxicología, Department of Pharmacology and Toxicology, School of Medicine, Department of Pharmacology and Toxicology, School of Medicine, University of Antioquia, Colombia,; 2 Hospital Infantil Concejo de Medellin, Medellin, Colombia

**Keywords:** Bronchiolitis, Colombia, global burden of disease

## Abstract

**Introduction:**

acute bronchiolitis is the leading cause of hospitalization in infants worldwide. However, little is known about the real impact of on society in terms of years of life lost due to this condition. The objective of the present study is to determine the Disability-Adjusted Life Years (DALYs) for acute bronchiolitis in infants in Colombia.

**Methods:**

data from the national epidemiological surveillance system were used to estimate DALYs, calculated from the sum of years of life lost and years lived with disability due to acute bronchiolitis in Colombia. A bootstrapped method with 10,000 iterations was used to estimate each statistical parameter using the package DALYs calculator in R.

**Results:**

in 2019, 447,434 years of life (confidence interval 95% 397,647- 512,759) were lost due to acute bronchiolitis in Colombian infants. The estimated rate was 34 DALYs/1000 person-year (95% confidence interval 30-39).

**Conclusion:**

our paper shows the high burden of disease associated with bronchiolitis in Colombia. Prevention strategies, such as acute bronchiolitis vaccination, to reduce morbidity associated with acute bronchiolitis should be encouraged in our country.

## Introduction

Acute bronchiolitis is a leading cause of hospitalization in pediatric patients [1]. Classical measures of disease as mortality and incidence does not give an entire representation of the burden of disease produced by individuals in different populations. The burden of disease is evaluated using the disability-adjusted life year (DALYs), a time-based measure that link years of life lost due to premature mortality (YLL) and years of life lost due to time lived in states of less than full health, or years of healthy life lost due to disability (YLD). One DALYs means the loss of the equivalent of one year of full health [2]. Our group in Colombia estimated 260,873 years of life (CI95% 208,180-347,023) were lost due to respiratory virus for respiratory syncytial virus , the most frequent cause of bronchiolitis, in children under 2 years in 2019 [3]. However, this study does not estimate it directly for acute bronchiolitis because up to 40% of cases may be due to viruses other than respiratory syncytial virus [1]. A valid and consistent description of the burden of disease is a great input to generate better health-policies and planning processes. Here, we estimated the disease burden of acute bronchiolitis in infants in Colombia.

## Methods

**Study design**: using the methods described by Murray and Lopez [2], we estimated the DALYs for acute bronchiolitis infection. DALYs were calculated for the most important health outcomes of this infection: acute bronchiolitis no complicated, acute bronchiolitis with acute mild or moderate complications (hypoxemia, atelectasis, and pneumonia), acute bronchiolitis with severe acute complications (PICO admission, pneumothoraxes, pleural effusions, sepsis) and acute bronchiolitis with long term complications (recurrent wheezing). The study protocol was reviewed and approved by the Institutional Review Board of the University of Antioquia (No 18/2015).

**Data sources and model parameters**: to estimate the burden of disease we use incidences and mortality rates from comprehensive data reported by the national epidemiological surveillance system during 2019 [4]. The mortality data was validated with the data reported by the National Department of Statistics during the same time [2]. Informed consent was not required because we used surveillance data without personal identifiers. To estimate the ranges of incidence and mortality rates, as well as and guarantee the external validity in the sensitivity analysis, this information was supplemented with search for studies previously published with Colombian patients [5-10]. The years of life lost by premature mortality were estimated, per outcome, by multiplying the number of deaths due to this outcome -in infants with acute bronchiolitis- by the number of years of expected remaining life at the age of death according to reference life tables of the global burden of disease study as recommended the manual of GBD studies [2]. All estimates used the Colombian population in 2019. Next, the YLD per outcome was obtained by multiplying the number of cases -per outcome in infants with acute bronchiolitis-by both: the average duration of this outcome obtained from the literature [5], and respective disability weight derived from the 2015 GBD study (0.051 for acute bronchiolitis no complicated or with acute mild or moderate complications, 0.13 for acute bronchiolitis with severe acute complications and 0.06 for acute bronchiolitis with long term complications) (Table 1) [4,6,8-10].

**Table 1:** model inputs: morbidity probabilities used in the base case and sensitivity analyses [4,6,8-10]

Model input	Cases/1000 persons/year			
	**Mean**	**Min**	**Max**	**Source**
Incidence of bronchiolitis without acute complications				[4,9]
0 to 12 months	63,7	53,1	70,2	[4,9]
13 to 24 months	34,7	30,2	40,3	[4,9]
Mortality of bronchiolitis without acute complications				[4,9]
0 to 12 months	0.31	0.25	0.4	[4,9]
13 to 24 months	0.10	0.05	0.15	[4, 9]
Incidence of bronchiolitis with mild or moderate acute complications	2,79	2,50	3,07	[4,9]
Mortality of bronchiolitis with mild or moderate acute complications	0.57	0.55	0.61	[4,9]
Incidence of bronchiolitis with severe complications	1,08	0,87	1,91	[9,10]
Mortality of bronchiolitis with severe complications	0.13	0.10	0.45	[9,10]
Incidence of bronchiolitis with long term complications	20,20	15,2	2,02	[6,8]
Mortality of bronchiolitis with severe complications	0.06	0.06	0.26	[6,8]

**Statistical analysis**: the internal consistency of each parameter was evaluated using the DISMOD II program following the recommendations of manual for national studies of the WHO disease burden [2]. To estimate the confidence Interval around YLD, YLL, and DALYs calculated before, we made 10 000 iterations in a Monte Carlo simulation, using a bootstrapped technique of DALYs calculator package in R to obtain each confidence interval, with a discount rate of 3% and weighting by age [2]. The DALYs was expressed both in absolute value and per 1,000 person-years. Multi-way probabilistic sensitivity analysis was made using the standardized regression coefficient method [11].

## Results

In 2019, we estimated that ~447,434 years of life (confidence interval or CI 95% 397,647- 512,759) were lost due to acute bronchiolitis infections in infants, with an estimated rate was 34 DALYs/1000 person-year (95% CI 30-39). Fifty-one percent (51%) (240,374 DALYs) were occurred in male infants. Sixty-one percent (61%) (290,800 DALYs) of affected infants between 1 to 2 years of age (Table 2). Ninety-two percent (92%) of DALYSs represented years of life lost due to early death, and 8% years of healthy life lost due to disability. The estimated rate of acute bronchiolitis with acute mild or moderate complications was 19 DALYs/1,000 person-year (CI 95% 18.5-19.5), for acute bronchiolitis with severe acute complications 6.3 DALYs/1000 person-year (CI 95% 4.3-10), for acute bronchiolitis with long term complications 5.2 DALYs/ 1,000 person-year (CI 95% 2.5-9.7) and for uncomplicated acute bronchiolitis: 5.9 DALYs/1000 person-year (CI 95% 5.9-6.6). Most of DALYs in the health outcomes happen between 12 to 24 months, except in uncomplicated acute bronchiolitis. The results were robust in the sensitivity analysis. The percentage of change in the total estimate of DALYs did not exceed 25% with the variables analyzed; being the probability of death the variable associated with the highest percentage of change in the DALYs (between 5-25%, of the final estimate). There were no significant variations in the discount rate between 0 to 5%.

**Table 2:** distribution by sex and age of disability-adjusted life years

	DALYSS	YLD	YLL			
Age	**Men**	**Female**	**Men**	**Female**	**Men**	**Female**
0 - 1 year	91, 706	87,504	4346	4147	87,360	83,357
1- 2 year	148,668	142,132	8582	8205	140,086	133,927

DALYs: disability-adjusted life years; YLD: years lived with disability; YLL: years of life lost)

## Discussion

We found that acute bronchiolitis in infants generates a significant number of years of life lost highlighting not only the importance of this etiological agent but also the usefulness of using DALYs to assess the true weight of a disease in society. The national burden study estimated In Colombia for LTRI around 3.98 DALYs per 1000 in infants [12]. Our estimate was higher (was 37 DALYs/1,000 person-year) because the national burden of disease study used data from national health surveys while we examined the records of epidemiological surveillance. Our records have a greater degree of completeness since they are mandatory in Colombia.

Otherwise, if acute bronchiolitis alone generates 37 DALYs for 1,000 infants, this disease would be only behind of low birth weight in the total estimation of DALYs in this age group [12]. When we compare our results with other estimations of DALYs of different diseases, acute bronchiolitis in infants, generates more years of life lost than cervical cancer between 45-59 years (1.6 DALYs per 1,000 inhabitants), epilepsy between 30-44 years (1 DALYs per 1,000 inhabitants) and leukemia in infants between 5-14 years (1 DALYs per 1,000 inhabitants) [12]. This highlights the importance of generating specific burden of the disease studies by etiological agent, but also that it should encourage the development of vaccines, which according to our estimates would have a high population impact. Burden of disease studies should be a primary source for prioritization exercises in public health. Although in our continent even the use of health technology assessment and advanced statistical information is not the main input for decision-making, this type of estimations such as ours should encourage decision-makers to use evidence to make health decisions [13-15].

This study has limitations. First, we may have some degree of information bias and underestimation due to the use of data from the national surveillance and notification system. However, LRTI cases have florid symptomatology in this age group, often prompting medical attention. There are a global increasing in the reporting of cases to SIVIGILA has been noted [4], and in our sensitivity analysis, the final result of DALYs was not sensitive to the change in values of these probabilities, guaranteeing the robustness of the model. There are no specific “disability weights” for acute bronchiolitis infection. In this case, we used data reported for LRTI because in terms of mortality it does not differ from data presented by patients with other viruses in Colombia. In the sensitivity analysis, the percentage of change in the total estimate of DALYs did not exceed 25% within the variables analyzed.

## Conclusion

The burden of acute bronchiolitis is a serious problem in Colombia, with a considerable social impact in terms of disability and mortality. Morbidity and mortality rates can be improved not only by effective prevention and promotion of public policies but also by improvements in the quality of health care services. Our results prompt evaluation of public health interventions and novel biological preventive strategies under evaluation to minimize the impact of this serious problem in Colombian infants.

### What is known about this topic

- Acute bronchiolitis is the leading cause of hospitalization in infants worldwide;
- Despite that, previous studies had estimated the burden of disease of lower respiratory tract infections, in term of years of life lost by premature death or disability, theses report do not estimate it directly for acute bronchiolitis.

### What this study adds

- We found that acute bronchiolitis in infants less than 2 years generates a significant number of years of life lost;
- Prevention strategies, such as acute bronchiolitis vaccination to reduce morbidity associated with acute bronchiolitis should be encouraged in our country.
